# Use of an Online Ultrasound Simulator to Teach Basic Psychomotor Skills to Medical Students During the Initial COVID-19 Lockdown: Quality Control Study

**DOI:** 10.2196/31132

**Published:** 2021-11-01

**Authors:** Jean-Yves Meuwly, Katerina Mandralis, Estelle Tenisch, Giuseppe Gullo, Pierre Frossard, Laura Morend

**Affiliations:** 1 Department of Radiology University Hospital of Lausanne University of Lausanne Lausanne Switzerland; 2 Medical Pedagogy Unit University of Lausanne Lausanne Switzerland

**Keywords:** anatomy, computers in anatomical education, internet application in anatomy, medical education, ultrasonography, ultrasound, simulation, simulator, psychomotor, motor skills, medical students, teaching

## Abstract

**Background:**

Teaching medical ultrasound has increased in popularity in medical schools with hands-on workshops as an essential part of teaching. However, the lockdown due to COVID-19 kept medical schools from conducting these workshops.

**Objective:**

The aim of this paper is to describe an alternative method used by our medical school to allow our students to acquire the essential psychomotor skills to produce ultrasound images.

**Methods:**

Our students took online ultrasound courses. Consequently, they had to practice ultrasound exercises on a virtual simulator, using the mouse of their computer to control a simulated transducer. Our team measured the precision reached at the completion of simulation exercises. Before and after completion of the courses and simulator’s exercises, students had to complete a questionnaire dedicated to psychomotor skills. A general evaluation questionnaire was also submitted.

**Results:**

A total of 193 students returned the precourse questionnaire. A total of 184 performed all the simulator exercises and 181 answered the postcourse questionnaire. Of the 180 general evaluation questionnaires that were sent out, 136 (76%) were returned. The average precourse score was 4.23 (SD 2.14). After exercising, the average postcourse score was 6.36 (SD 1.82), with a significant improvement (*P*<.001). The postcourse score was related to the accuracy with which the simulator exercises were performed (Spearman rho 0.2664; *P*<.001). Nearly two-thirds (n=84, 62.6%) of the students said they enjoyed working on the simulator. A total of 79 (58.0%) students felt that they had achieved the course’s objective of reproducing ultrasound images. Inadequate connection speed had been a problem for 40.2% (n=54) of students.

**Conclusions:**

The integration of an online simulator for the practical learning of ultrasound in remote learning situations has allowed for substantial acquisitions in the psychomotor field of ultrasound diagnosis. Despite the absence of workshops, the students were able to learn and practice how to handle an ultrasound probe to reproduce standard images. This study enhances the value of online programs in medical education, even for practical skills.

## Introduction

### Background

Ultrasound in medical education is gaining more and more interest due to the motivation of medical students on one hand [1] and to the increasing accessibility of ultrasound machines on the other [2,3].

Ultrasound teaching is classically divided into two mains parts: theoretical lectures focused on physics, image acquisition, indications, limitations and hygiene, and practical exercises focusing on the acquisition of psychomotor skills necessary to get diagnostic ultrasound images [4]. Until today, this practical part has been an essential element in the teaching of ultrasound [5]. In the medical school of the University of Lausanne, Switzerland, these practical ultrasound skills are usually acquired in normal circumstances, during the ultrasound workshops set up by the Radiology Department during the first year of master’s studies. These workshops combine practical work on ultrasound machines with tutors and practice on a simulator. Additional optional hands-on workshops offered by the Young Sonographers (a group of students teaching ultrasound to other students) aim to complement these practical skills [6]. Primarily, these courses focus on the learning of normal anatomy in ultrasound.

In 2020, the COVID-19 pandemic abruptly disrupted the habitual working/learning practices of many medical entities. The impact was felt from emergency departments [7,8] to interventional radiology [9]. Courses in our medical school were also affected by the pandemic as early as mid-March 2020, since live courses were annulled and students were prevented from attending the workshops. To fill this gap, we have turned to an alternative solution with the establishment of a complete online ultrasound course.

This method of teaching ultrasound has been structured into two parts. First, to make a detailed presentation of the ultrasound exploration of the different systems and to complete the theoretical teaching of the bachelor’s years, we directed the students to the “Blended learning” site of the Institute of General Medicine of the University of Bern [10,11]. Five different modules were accessible for the students on this website to self-teach the basic foundations of medical ultrasound. The first module was dedicated to ultrasound physics and the manipulations of an ultrasound machine. The next modules focused on the ultrasound exploration of normal anatomy in the following order: thoracic and abdominal organs, the musculoskeletal system, and finally the lymph nodes and cervical organs. All modules dedicated to normal anatomy were organized in the same way: presentation of a clinical case, description of the normal sonographic anatomy, video demonstration of the sonographic exploration of the organ, description of tips and tricks, and finally solution of the clinical case. The courses offered on this website are part of the “Young Sonographers” training course for students in Swiss medical schools to validate the Basic Course in Abdominal Ultrasound at the Swiss Society of Ultrasound in Medicine (SSUM-SGUM). Second, the students were asked to carry out a series of practical exercises dedicated to learning psychomotor skills for the practice of ultrasound that is accessible on an online simulator.

### Objectives

The aim of this paper is to describe and evaluate this alternate online method we used to train our medical students in the acquisition of the visuospatial and visuomotor skills required to manipulate an ultrasound transducer and produce images of diagnostic quality without live workshop attendance.

## Methods

**Figure 1:**
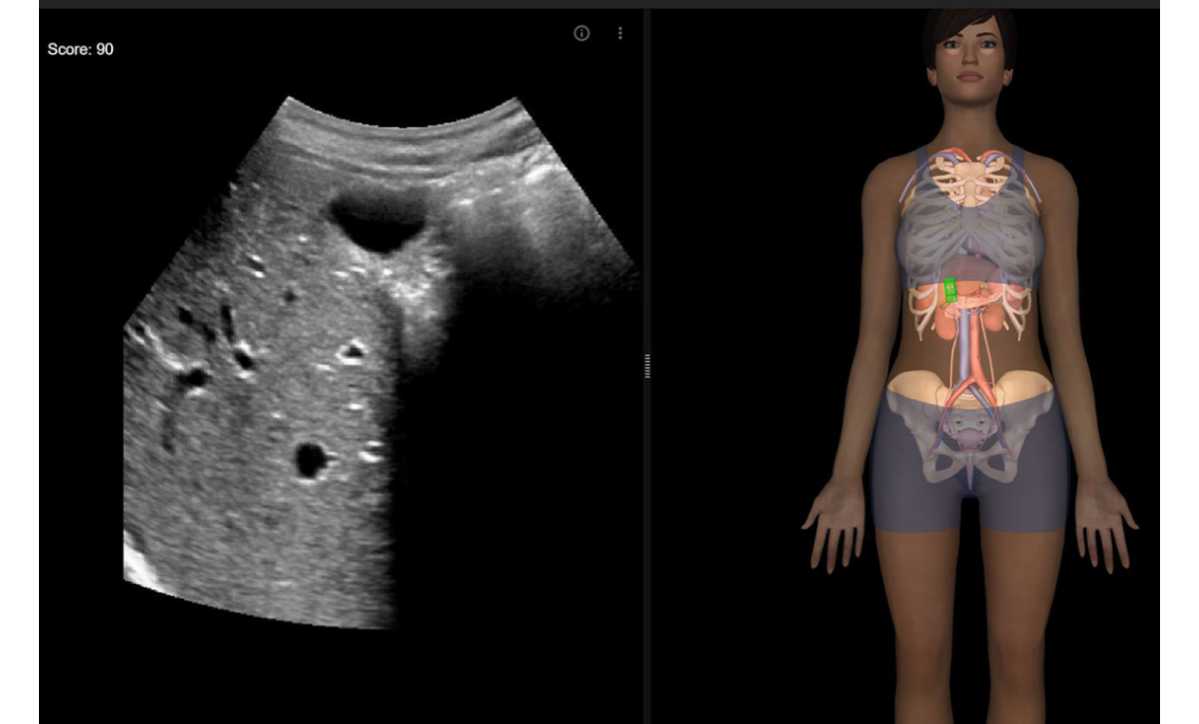
View of the simulator interface, with the generated ultrasound slice on the left and the probe on the mannequin on the right. As the probe is drawn closer to the reference position, it becomes green. At the same time, the counter (at the top, on the left) increases up to 100 (perfect matching).

The online simulator was provided by an Israeli start-up (Innoging, Tel Aviv; Figure 1). This start-up provided us with an empty virtual mannequin that we filled with volumes of ultrasound images acquired on site. These volumes of images were acquired from ultrasound exploration of a volunteer with a machine used for daily clinical work. We proceeded by manually sweeping an ultrasound probe (either convex low-frequency 1-5 MHz for abdominal images or linear high-frequency 4-18 MHz for superficial images) on the different regions of interest (liver, gallbladder, abdominal vessels, spleen, kidneys, bladder, uterus and ovaries, thorax, thyroid, axillary lymph nodes, groin, and Achilles’ tendon). Video clips that were 20 seconds were obtained, each containing 350 images. One sweep along the short axis of the probe gave one volume in video/DICOM format. In that way, 30 volumes were produced and were sent through the internet to the simulator provider, which created a library of available volumes. We then placed the different volumes, by choosing in that library, at the right location in the virtual mannequin. From these different ultrasound volumes, we developed a series of 56 practical exercises related to the chapters of the “Blended learning” courses (bases of probe manipulation, large vessels, spleen, thorax, liver, pancreas, gallbladder, bile ducts, kidneys, urinary tract, bladder, uterus, musculoskeletal system, neck, lymph nodes; Textbox 1). Each exercise had the same structure: first, a brief comment on the structure to follow when scanning, the presentation of the image to reproduce, the place to store the reproduced image, and finally the initial image again with anatomical captions.

**List of the different simulation exercises with their connection to the theoretical chapters of the “Blended learning.”**

Bases of probe manipulation

- Liver and right kidney
- Bladder and uterus
- Heart
- Pregnancy
- Right kidney
- Liver and gallbladder

Large vessels, spleen, thorax

- Aorta
- Aorta
- Aorta and inferior vena cava (IVC)
- Aorta and IVC
- IVC
- Spleen
- Spleen
- Spleen
- Spleen
- Thorax
- Thorax
- Thorax
- Thorax

Liver, pancreas, gallbladder, bile ducts

- Liver
- Liver
- Liver
- Liver and gallbladder
- Liver
- Liver and main biliary duct
- Liver and main biliary duct
- Gallbladder
- Gallbladder
- Gallbladder
- Left liver
- Pancreas
- Pancreas
- Pancreas

Kidneys, urinary tract, bladder, uterus

- Right kidney
- Right kidney
- Left kidney
- Left kidney
- Left kidney
- Right kidney
- Bladder
- Uterus
- Bladder and uterus
- Bladder and uterus
- Bladder and uterus

Musculoskeletal system, neck, lymph nodes

- Achilles’ tendon
- Achilles’ tendon
- Thyroid
- Thyroid
- Thyroid
- Cervical lymph nodes
- Cervical lymph nodes
- Thyroid
- Thyroid
- Cervical lymph nodes
- Groin
- Groin

Every student in the first year of master’s studies in the medical school of the University of Lausanne, Switzerland received a personal code to access the simulator. They had to carry out the various exercises proposed, with the recommendation of practicing the exercises only after studying the corresponding chapter of the “Blended learning” course. Access was open from mid-May to the end of August 2020.

The completion of exercises was monitored. The precision obtained by the trainee during the simulator exercises, where a typical image had to be reproduced, was measured as a percentage of the reference position given at the beginning of each exercise.

**Figure 2:**
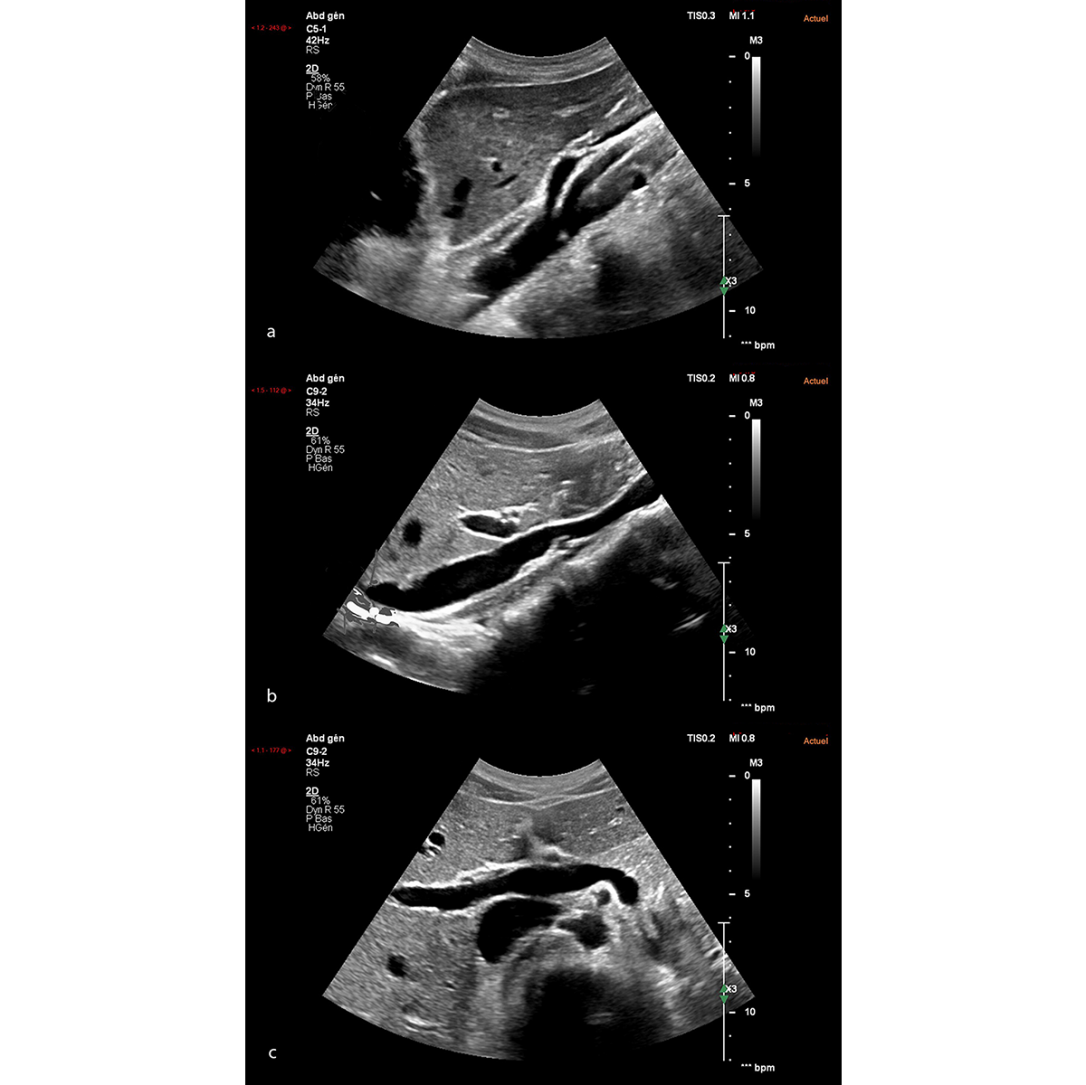
Example of a question of the pre-posttest: I have just got the previous images (images 2a and 2b). To get the image 2c during the same run, I have to make the following movement with my probe: (a) translation to the left, (b) anticlockwise rotation, (c) inclination to the right, (d) I don't know. Answer b: anticlockwise rotation.

A questionnaire targeting the manipulation of an ultrasound probe had to be completed by each participant at the beginning and at the end of the training (pre- and postcourse, 20 questions, maximum score 10; Figure 2). In addition, as with the other faculty courses, a final general assessment questionnaire was submitted to the students, using a qualitative scale. Students did not receive the results, neither at the beginning (pretest) nor at the end (posttest) of the educational process.

The study was designed as a quality control for ultrasound teaching. As such, our ethical review board determined that the project did not fall within the scope of the Law on Human research (Req-2021-00589), and informed consent was waived. The data was anonymized to guarantee the privacy of participants.

The statistical analyses were carried out with *t* test, Wilcoxon rank sum test, or Spearman test on the STATA software (version 16.1, StataCorp). A *P* value<.05 was considered significant.

## Results

A total of 193 students finished the precourse training during the allotted period. A total of 184 students performed all the simulator exercises and 181 performed the postcourse test. The faculty sent 180 evaluation questionnaires, of which 136 (76%) were returned.

**Figure 3:**
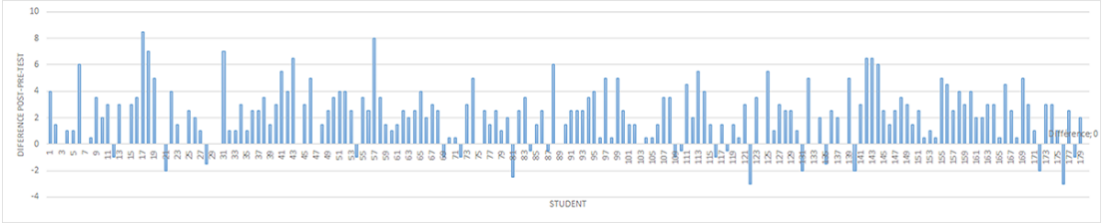
Differences between the posttest score and pretest score. The mean difference was 2.12 (SD 2.13), with a maximum of 8.5 and a minimum of –3. The comparison of the two tests showed a significant difference, with an improvement in performance after the simulation exercises (Wilcoxon rank sum test; *P*<.001). Note that for 20 students, the posttest score was worse than the pretest score.

**Figure 4:**
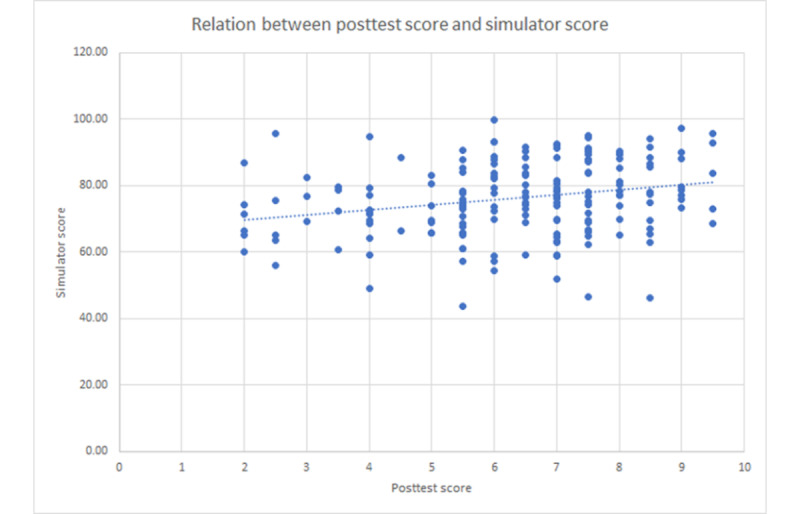
Relation between the simulator score (precision of the execution of the simulation exercises) and the posttest score (understanding of probe manipulation) for each student (Spearman rho 0.2664; *P*<.001).

Analysis of the target questionnaires determined that the scores had increased significantly after the completion of the simulator exercises (Figure 3). The mean precourse score was 4.23 (SD 2.14), whereas after completion of the exercises, it was 6.36 (SD 1.82; *P*<.001). The postcourse score for all students was related to the accuracy with which the simulator exercises were performed (Spearman rho 0.2664; *P*<.001; Figure 4). On the simulator, the lowest score was 43.68, the highest 99.67, with a mean of 76.11 (SD 11.29).

A total of 20 students performed better during the precourse (mean score 6.75, SD 1.93) than during the postcourse (mean score 5.32, SD 1.97; *P*<.001). There was a nonsignificant difference between the precourse score of these students and the mean postcourse score including all students (*P*=.38). There was no significant difference between the simulator score of that group of students (mean 76.69, SD 10.66) and the score of all students (mean 76.11, SD 11.29; *P*=.70).

Nearly two-thirds (n=84, 62.6%) of the students said they enjoyed working on the simulator. A total of 79 (58.0%) students felt that they had achieved the course’s objective of reproducing ultrasound images. A total of 115 (84.5%) estimated that, after that course, they were able to recognize the abdominal organs by ultrasound. A total of 83 (61.9%) students found that the exercises were adapted to their previous knowledge, and 68.6% (n=92) felt that they had progressed in understanding ultrasound anatomy. Inadequate connection speed had been a problem for 40.2% (n=54) of students.

Among the negative comments, difficulty in manipulation of the simulator probe and the mannequin from the computer mouse or trackpad were frequently cited. Another recurring remark was related to the “Blended learning” courses being in the German language.

## Discussion

### Principal Findings

Our study has demonstrated that the integration of an alternative teaching model during the pandemic lockdown for the practical learning of ultrasound with an online mode has allowed substantial psychomotor skill acquisitions for ultrasound diagnosis.

The COVID-19 pandemic has impacted medical education everywhere. Universities were forced to switch to online teaching [12]. These online methods, such as lectures, videos on websites, or webinars, are primarily focused on theoretical knowledge. The online teaching of clinical competencies or skills, particularly ultrasound, has not been reported in the literature to date. To our knowledge, we present here the largest study evaluating the effects of an online ultrasound simulator on the psychomotor performance of medical students. This is also the first report of a completely autonomous self-learning program for basic ultrasound learning [13].

Despite the limitations related to the urgency of its implementation (course of “Blended learning” in a foreign language, difficulties in manipulating the probe of the simulator with a mouse or trackpad, difficulties in orienting the mannequin, simulation with slow internet connection) nearly two-thirds of students were satisfied with the experience.

Simulation is a popular teaching method in health care education [14]. During simulation exercises, students may indefinitely repeat gestures and procedures without any influence on clinical workload or patient safety [15,16]. Many different kinds of ultrasound simulators are available on the market [17]. Some require a consecrated computer, a mannequin, and a fake probe; others only need a fake transducer with a downloadable software, or others are completely dematerialized on the web [18,19].

Whatever the technology and the price of the ultrasound simulator, many reservations are expressed about the real place of simulation in ultrasound teaching [13,20]. The main criticism rests on the scarcity of scientific studies analyzing the real impact of simulation-based ultrasound training on clinical practice. Indeed, the majority of available studies either analyze the effect of simulator training on the simulation experience [21,22] or use subjective evaluations to support their conclusions [23-26].

For 20 students, the posttest score was lower than the pretest score. However, their score on the simulator was the same as that of all the students.

Some students had already received an introduction to ultrasound before the project was launched. We did not specifically ask the question of previous training to the participants. Nevertheless, a closer look at this group of “negative” performers revealed that it consisted of many pupils repeating the grade and young sonographers and students in higher grades from other universities; in other words, students with previous exposure to ultrasound training.

Students had free access to the new learning method during the 3.5 months. They received the initial instructions and then they were free to proceed to the activities at their own pace and convenience. As for other medical students worldwide, lockdown did not mean enforced idleness. They were committed to different health care initiatives against COVID-19 [27]. As a result, they proceeded with the requested activities only when they had time during their busy schedule. That may explain the surprising results of the group of “negative” performers: at the beginning of the educational program, they had conscientiously fulfilled the pretest questionnaire and then carefully carried out the different exercises on the simulator. At the end of the process, as they had already experimented on this method and under the pressure of their other activities, motivation for repeating and for the completion of the questionnaire dropped. Indeed, motivation in medical education is important [28], particularly in a self-learning environment [29].

Difficulties with manipulations of the simulator probe and the mannequin from the computer mouse or trackpad were mentioned as a problem in the simulation’s experience. Gunabushanam et al [30] have recently proposed a smart solution for the virtual ultrasound simulator to resemble the ultrasound transducer: a user’s own smartphone [30]. Such a solution has the potential to give the realistic touch needed for a complete virtual ultrasound simulation experience. Interestingly, the main suggestion for improvement proposed by the group of users of the smartphone’s mock probe was to add an option allowing to scroll through images with a mouse [30].

### Limitations

Unfortunately in our study, we could not evaluate the impact of online simulation training on real practical skills. Because of the completely virtual setup of the study, only simulator-based analysis and subjective feedback were available for analysis. A future part of the study will compare the ultrasound scanning performance of different groups of students: the young sonographers’ tutors, the medical students exposed to the simulator without additional supervised training, and the young sonographers’ students with traditional training. This later group is still in training with hands-on workshops. As soon as the present lockdown will be lifted, we will proceed with this additional study. Another limitation was related to the foreign language of the “Blended learning” course. This course is a part of the official program of medical students’ ultrasound teaching from the SSUM-SGUM. The translation in the other official Swiss languages has been planned, but as with many other projects this year, it has been delayed because of the pandemic.

### Conclusions

We have observed that exposition of medical students to a completely virtual ultrasound simulation experience has enabled them to acquire substantial visuospatial and visuomotor ultrasound skills. Until today, these competencies were only acquired through practical work on site with tutors. With this study, we demonstrated the value of a dedicated online method to remotely teach practical ultrasound. Self-learning has the potential of substantially reducing the barriers to use ultrasound simulation and its potential costs.

## Abbreviations

SSUM-SGUM: Swiss Society of Ultrasound in Medicine
